# Correction: Adsorption properties of NO molecules on the hexagonal LaCoO_3_ (0 0 1) surface: a density functional theory study

**DOI:** 10.1039/c8ra90059k

**Published:** 2018-07-17

**Authors:** Zhijie Liu, Yanxin Wang, Hongwei Gao

**Affiliations:** Key Laboratory of Plant Resources, Chemistry in Arid Regions, Xinjiang Technical Institute of Physics and Chemistry, Chinese Academy of Sciences Urumqi 830011 China gaohongw369@ms.xjb.ac.cn +86-991-3858319 +86-991-3858319

## Abstract

Correction for ‘Adsorption properties of NO molecules on the hexagonal LaCoO_3_ (0 0 1) surface: a density functional theory study’ by Zhijie Liu *et al.*, *RSC Adv.*, 2017, **7**, 34714–34721.

The authors regret that the funder information in the Acknowledgements section is incorrect in the original article. The correct information is shown below.

This study was supported by the Natural Science Foundation of Xinjiang, China, Grant No. 2016D01A073.

The Royal Society of Chemistry apologises for these errors and any consequent inconvenience to authors and readers.

## Supplementary Material

